# Comparison and Outcome Analysis of Patients with Takotsubo Cardiomyopathy Triggered by Emotional Stress or Physical Stress

**DOI:** 10.3389/fpsyg.2017.00527

**Published:** 2017-04-27

**Authors:** Konstantinos Giannakopoulos, Ibrahim El-Battrawy, Katja Schramm, Uzair Ansari, Ursula Hoffmann, Martin Borggrefe, Ibrahim Akin

**Affiliations:** ^1^First Department of Medicine, Faculty of Medicine, University Medical Centre Mannheim, University of HeidelbergMannheim, Germany; ^2^German Center for Cardiovascular Research (DZHK), MannheimGermany

**Keywords:** takotsubo cardiomyopathy, physical stress, emotional stress, prognosis, mortality

## Abstract

**Background:** Previous studies revealed that takotsubo cardiomyopathy (TTC) is triggered by physical and emotional stresses. This study was performed to determine the short- and long-term prognostic impact of emotional- and physical stress associated with TTC.

**Methods and results:** Our institutional database constituted a collective of 84 patients diagnosed with TTC between 2003 and 2015. The patients were divided into two groups as per the presence of emotional stress (*n* = 24, 21%) or physical stress (*n* = 60, 52.6%). The endpoint was a composite of in-hospital events (thromboembolic events and life-threatening arrhythmias), myocardial infarction, all-cause of mortality, re-hospitalization due to heart failure, stroke, and recurrence of TTC. A Kaplan–Meier analysis indicated a significantly lower event-free survival rate over a mean follow-up of 5 years in the emotional group than the physical stress group (log-rank, *p* < 0.01). Multivariate Cox regression analysis revealed only emotional stress (HR 0.4, 95% CI: 0.2–0.9, *p* < 0.05) as a negative independent predictor of the primary endpoint.

**Conclusion:** Rates of in-hospital events and short- as well as long-term events were significantly lower in TTC patients suffering from emotional stress as compared to patients with physical stress.

## Introduction

Takotsubo or stress-induced cardiomyopathy, first described in the 1990s, is an acute reversible condition, characterized by a range of wall motion abnormalities. Takotsubo cardiomyopathy mimics a myocardial infraction and is clinically representative of an acute heart failure syndrome with substantial risk for adverse events (Akashi et al., 2008; Schneider et al., 2014; Stiermaier et al., 2015a,b; Becher et al., 2016; El-Battrawy et al., 2016).

Approximately 1–2% of all patients with acute coronary syndrome are eventually diagnosed with takotsubo cardiomyopathy (TTC) (Gianni et al., 2006; Kurowski et al., 2007). Most TTC patients are postmenopausal women (Elesber et al., 2006; Haghi et al., 2006; Jabara et al., 2009; El-Battrawy et al., 2016) and precipitating risk factors include the presence of emotional (reported in 27%) and physical stresses (reported in 38%). Other risk factors influencing the onset of a TTC event are smoking, alcohol abuse, anxiety states and psychiatric disorders (Templin et al., 2015), a smaller left ventricular size and hyperlipidaemia (Castillo Rivera et al., 2011; Zeb et al., 2011).

An early classification system based on the ballooning pattern of ventricular walls, as diagnosed by transthoracic echocardiography and levo-cardiography, enabled to sort patients into identifiable groups (Elesber et al., 2006; Haghi et al., 2006; Templin et al., 2015; El-Battrawy et al., 2016). The apical form is the most common (81.7%) followed by mid-ventricular (14.6%), basal (2.2%), and focal form (1.5%). The wall motion abnormalities do not usually correspond to a single coronary artery distribution and a coronary angiography need not necessarily suggest evidence of an acute occlusive coronary disease.

The underlying pathophysiological mechanism contributing to the evolution of TTC is poorly understood. There have been several hypotheses explaining the typical cardiac response and morphological appearance, however, these are yet to be conclusively confirmed. A leading hypothesis suggests that catecholamine surges accompanied by cellular calcium overload results in regional microvascular dysfunction in susceptible patients (Nef et al., 2009). The myocardial stunning supposedly caused by direct catecholamine-induced myocardial toxicity in TTC patients has been attributed to the ensuing diffuse catecholamine-induced microvascular spasm or dysfunction (Wittstein et al., 2005).

A preceding emotional or physical stress has been believed to be the trigger for most TTC patients. The present study was performed to define the prognostic impact of emotional versus physical stresses as a trigger factor of TTC, and furthermore, determining the long-term outcomes in these patients.

## Materials and Methods

Our single-center TTC patient database constituted a collective of 114 patients. A total of 84 of these patients were identified with potential emotional or physical triggers and consecutively observed in this study. Thirty patients with unclear stress triggers were excluded.

Physical stress was defined as the presence of acute medical illness, whereas emotional stress was defined by the detection of emotional stress (such as grief/loss, anxiety, financial problems, catastrophic medical diagnosis etc.), elicited through a detailed clinical history of the patient and precluding any form of acute illness.

Patients were diagnosed according to the Mayo Clinic Criteria (Madhavan and Prasad, 2010), which outlines the clinical features associated with TTC. The first criterion describes the transient wall motion abnormality in the left ventricular mid-segments with or without apical involvement; regional wall motion abnormalities that extend beyond a single epicardial vascular distribution; and frequently, but not always in the event of a stressful trigger. The second criterion stipulates the absence of obstructive coronary disease. The third criterion outlines the appearance of new ECG pathologies, which mimic ACS or modest elevations in cardiac troponin levels. The final criterion is the absence of pheochromocytoma and myocarditis in the patient. The patients were classified into two groups according to emotional and physical stresses, essentially outlined as the trigger of TTC. All angiograms, echocardiograms and ECGs were reviewed by two experienced independent cardiologists to evaluate the diagnosis of TTC.

This study was conducted in compliance with the Declaration of Helsinki concerning investigations in human subjects and the study protocol was approved by the Ethics Committee of University Medical Centre Mannheim.

In-hospital events such as arrhythmias, cardiac rupture, thromboembolic events, pulmonary congestion with the use of non-invasive positive-pressure ventilation, endotracheal intubation, use of inotropic agents, and in-hospital death etc. were assessed based on chart review. The primary end-point of our study was a composite of thromboembolic events, life-threatening arrhythmias, all-cause mortality, re-hospitalization due to heart failure, stroke, and recurrence of TTC as assessed by chart review and/or telephone review. If medical records, treating physicians or relatives were unable to provide further information concerning the circumstances of death, it was defined as death due to unknown cause.

### Statistics

The data is presented as means ± SD for continuous variables with a normal distribution, median (interquartile range) for continuous variables with a non-normal distribution, and as frequency (%) for categorical variables. The Kolmogorov–Smirnov test was used to assess normal distribution. Student’s *t*-test and the Mann–Whitney *U*-test were used to compare continuous variables with normal and non-normal distributions, respectively. The Chi-squared-test or Fisher’s exact test was used to compare categorical variables. The log-rank test was used to compare the survival curves between emotional versus physical stress group. Factors with *p* < 0.10 on univariate analysis were computed into a Cox multivariate regression to define independent risk factors for the end-point. Statistical analysis was performed with SPSS in all analyses and *p* ≤ 0.05 (two-tailed) was taken to indicate statistical significance.

## Results

Our analysis is based on 84 patients with a mean follow-up of 1529 ± 1121 days. Patients were classified into two groups according to the presence of emotional (*n* = 24, 28.5%) or physical stress (*n* = 60, 71.4%).

Baseline demographics for all study patients are shown in **Table 1**. The age of patients was similar in both groups (67.46 ± 8.7 years versus 67.6 ± 11.78 years), however, there was significant gender bias with female preponderance in the emotional stress group (95.8% vs. 78.3%, *p* < 0.05).

**Table 1:** Baseline characteristics of 84 patients initially presenting with takotsubo cardiomyopathy (TTC).

Variables	Emotional stress (*n* = 24)	Physical stress (*n* = 60)	*p*-value^∗^
**Demographics**			
Age, mean ± SD	67.46 ± 8.70	67.60 ± 11.78	0.95
Female, *n* (%)	23 (95.83)	47 (78.33)	**0.05**
Male	1 (4.16)	13 (21.66)	**0.06**
**Symptoms, *n* (%)**			
Dyspnoe	2 (8.33)	32 (53.33)	**<0.01**
Chest pain	17 (70.83)	22 (36.66)	**<0.01**
**Clinic parameter**			
Systolic BP, mmHg	135.80 ± 27.97	129.87 ± 31.79	0.46
Diastolic BP, mmHg	82.08 ± 13.97	76.05 ± 16.33	0.25
Heart rate, bpm	89.90 ± 26.05	107.31 ± 28.22	**0.02**
**ECG Data, *n* (%)**			
ST-segment elevation	6 (25.00)	16 (26.66)	0.87
Inversed T-Waves	20 (83.33)	53 (88.33)	1.00
PQ-interval	158.60 ± 26.27	155.46 ± 31.28	0.69
QTc (ms), mean ± SD	488.05 ± 57.91	477.24 ± 50.30	0.42
**Laboratory values, mean ± SD**			
Troponin I (U/L)	1.61 ± 1.24	4.04 ± 5.73	**0.06**
Creatine phosphokinase (U/L)	365.18 ± 938.50	957.14 ± 3562.46	0.44
Creatine phosphokinase myocard type	19.29 ± 18.98	37.61 ± 70.68	0.50
C-Reactive protein (mg/l)	13.71 ± 15.21	74.38 ± 97.54	**<0.01**
Hemoglobin	12.79 ± 1.41	11.73 ± 2.28	**0.04**
Creatinine (mg/dl)	0.97 ± 0.38	1.21 ± 0.75	1.00
**Echocardiography data, *n* (%)**			
LV EF %	38.67 ± 8.61	38.72 ± 9.60	0.98
LV EF% at follow-up	52.30 ± 1.70	54.1 ± 1.40	1.00
RV-involvement	5 (20.8)	14 (23)	1.00
Apical ballooning	17 (70.83)	42 (70.00)	0.88
Mitral regulation	13 (54.16)	27 (45.00)	0.44
Tricspid regulation	11 (45.83)	23 (38.33)	0.52
**Medical history, *n* (%)**			
Smoking	7 (29.16)	19 (31.66)	0.82
Diabetes mellitus	7 (29.16)	13 (21.66)	0.46
Obesity (BMI > 25 kg/m^2^)	8 (33.33)	11 (18.33)	0.18
Hypertension	15 (62.50)	33 (55.00)	0.53
COPD	2 (8.33)	14 (23.33)	0.13
Atrial fibrillation	4 (16.66)	11 (18.33)	1.00
Coronary artery disease	7 (29.16)	9 (15.00)	0.13
History of malignancy	2 (8.33)	10 (16.66)	0.49
**Drugs on admission, *n* (%)**			
Beta-blocker	10 (41.66)	15 (25.00)	0.18
ACE inhibitor	8 (33.33)	17 (28.33)	0.80
ARB	3 (12.50)	5 (8.33)	0.69
Digitalis	0 (0)	1 (1.66)	1.00
ASS	7 (29.16)	14 (23.33)	0.70
Anticoagulation	1 (4.16)	3 (5.00)	1.00


^∗^*p-values for the comparison between emotional and physical stress group; SD, standard deviation; ECG, electrocardiogram; EF, ejection fraction; BMI, body-mass-index, COPD, chronic obstructive pulmonary disease; ACE, Angiotensin-converting-enzyme; Angiotensin-receptor-blocker. Bold values mean *p* < 0.05.*

Patients presented more often with dyspnea in the physical stress group (8.5% vs. 53.3%, *p* < 0.05), whereas patients from the emotional stress group (70.8% vs. 36.7%, *p* < 0.05) complained primarily of chest pain.

A lab work-up suggested that C-reactive protein mean values (74.38 ± 97.54 mg/dl vs. 13.71 ± 15.21 mg/dl, *p* < 0.01) were greater among patients presenting with physical stresses. Additionally, these patients had higher Troponin-I values at the time of diagnosis. The echocardiographic parameters such as left ventricular ejection fraction, right ventricular involvement and valve regurgitation were similar in both groups.

As far as in-hospital events (**Table 2**) were concerned, the need for ventilation was more in the physical trigger group (12.5% vs. 46.7%, *p* < 0.05).

**Table 2:** In-hospital events and treatment strategy.

Variables	Emotional stress (*n* = 24)	Physical stress (*n* = 60)	*p*-value ^∗^
Life-threatening arrhythmia	1 (4.16)	8 (13.33)	0.43
NPPV and intubation	3 (12.50)	28 (46.66)	**<0.01**
Inotropic agents	2 (8.33)	14 (23.33)	0.13
Resuscitation	1 (4.1)	6 (10)	0.66
Defibrillator-Implantation	1 (4.16)	0 (0)	0.28
VA-ECMO	0 (0)	1 (1.6)	1.00
Admission to ICU, length of stay	2.83 ± 2.01	6.02 ± 8.28	**0.06**
In-hospital death	0 (0)	6 (10)	0.17
Thromboembolic events	1 (4.16)	10 (16.66)	0.16
Acquired Long QTs	13 (54.16)	41 (68.33)	0.27
Cardiogenic Shock	2 (8.33)	14 (23.33)	0.20


^∗^*p-values for the comparison between emotional and physical stress group; NPPV, non-invasive positive pressure ventilation; VA-ECMO, veno-arterial extracorporal membrane oxygenation; ICU, Intermediate care unit. Bold values mean *p* < 0.05.*

**FIGURE 1:**
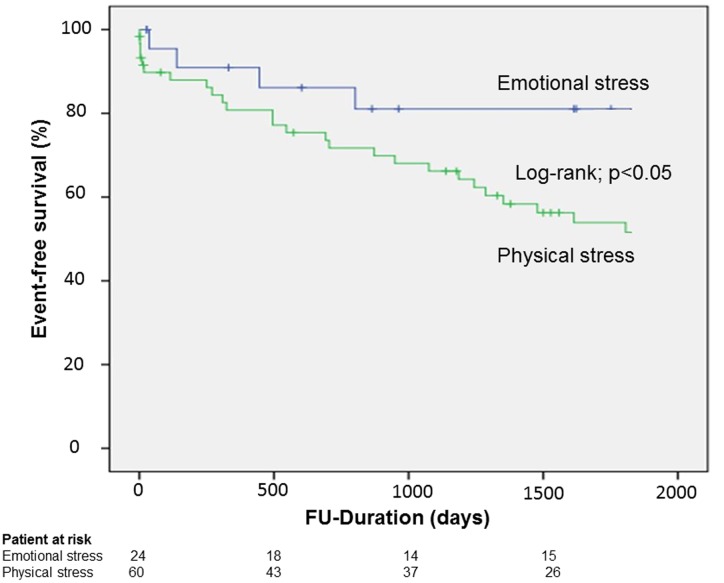
**Survival in patients suffering from takotsubo cardiomyopathy (TTC) with emotional stress compared to patient with physical stress**.

The Kaplan–Meier analysis showed significantly lower event-free survival rates in the physical stress group; log-rank *p* < 0.05 (**Figure 1**). A univariate analysis revealed that male gender, apical ballooning, EF ≤ 35%, atrial fibrillation and history of cancer were positive predictors of the composite endpoint and that emotional stress was a negative predictor of the composite endpoint. In the multivariate Cox regression analysis, male gender (HR 2.5, 95% CI 1.1–5.4; *p* = 0.02), atrial fibrillation (HR 2.08, 95% CI 1.0–4.2; *p* = 0.04), EF ≤ 35% (HR 1.79, 95% CI 0.9–3.3; *p* = 0.07) were positive independent predictors of the endpoint and it was only emotional stress that constituted a negative independent predictor of the endpoint (HR 0.43, 95% CI: 0.2–0.9), **Table 3**.

**Table 3:** Multivariate analysis for the composite endpoint.

	Univariate analysis			Multivariate analysis		
	HR	95% CI	*p*-value	HR	95% CI	*p*-value
Age	1.01	0.9–1.0	0.30			
Male	2.28	1.1–4.5	**0.01**	2.51	1.1–5.4	**0.02**
Coronary artery disease	1.44	0.8–2.7	0.25			
Apical ballooning	1.85	0.9–3.7	**0.07**	1.54	0.7–3.1	0.23
EF ≤ 35%	2.54	1.4–4.4	**<0.01**	1.79	0.9–3.3	**0.07**
Atrial fibrillation	2.00	1.1–3.6	**0.02**	2.08	1.0–4.2	**0.04**
Emotional stress	0.42	0.2–0.9	**0.02**	0.43	0.2–0.9	**0.03**
Physical stress	1.52	0.8–2.9	0.15			
Hypertension	0.92	0.5–1.6	0.78			
Diabetes mellitus	1.10	0.6–2.0	0.74			
History of cancer	2.23	1.2–4.3	**0.01**	1.03	0.4–2.3	0.96


*HR, hazard ratio; EF, ejection fraction. Bold values mean *p* < 0.05.*

## Discussion

We performed a retrospective clinical investigation in 84 consecutive TTC patients, and showed that (i) physical stress is more common than emotional stress as a possible trigger of TTC; (ii) the in-hospital morbidity and mortality rates were significantly higher in TTC patients with physical stresses; (iii) the long-term prognosis was poorer in patients associated with a physical trigger.

Takotsubo cardiomyopathy, first described in the Japanese population, occurs predominantly in postmenopausal women and the disease is usually provoked by emotional or physical stress. An enhanced sympathetic activity with elevation of catecholamine levels has been documented in these patients (Wittstein et al., 2005). Nevertheless, a defining explanation to its’ underlying pathogenesis remains unresolved.

Although TTC is now encountered as a benign disease, the in-hospital mortality has been reported to be as high as 1–4% of the affected population. Moreover, other TTC related complications such as congestive heart failure, cardiogenic shock, respiratory distress, and lethal arrhythmias have also been well reported (Schneider et al., 2014; Becher et al., 2016; El-Battrawy et al., 2016).

In our study-population, TTC was much more common in women than men and occurred in patients with a mean age of 67 years; findings that are in concordance to international literature. Additionally, male TTC patients were almost exclusively insulted by physical stresses, a result that could reaffirmed by other recently conducted studies (Schneider et al., 2013). Research discussing the incidence of physical and emotional stresses contributing to TTC have yielded variable results (Templin et al., 2015; Vizzardi et al., 2015). Physical stress was detected as a cause of TTC more often than emotional triggers among our patients (71.4%). The discrepancy between these results may be associated with the fact that a variety of factors initiate TTC and many of these triggers could fail detection during the initial clinical evaluation of the patient. Nevertheless, there has been consistent evidence claiming a high incidence of physical stress contributing to TTC in a tertiary center like ours (Lee et al., 2010). The differences in incidence, among a population of patients with neurological or psychiatric disorders, conditions that predispose to TTC (Gianni et al., 2006; Kurowski et al., 2007; Templin et al., 2015), may also play a role in these results.

In our TTC cohort, patients with physical stress induced TTC had higher values of c-reactive protein (CRP) levels. Although CRP is a non-specific indicator of an acute phase of disease, its value may be influenced by many clinical parameters. This statistical finding could indicate that the intensity of acute phase has a role in the pathogenesis of the syndrome and may predispose the patient to several complications (Morel et al., 2009; Song et al., 2012). There is evidence that in an acute myocardial infraction, higher acute phase proteins can predict the clinical outcome (Reindl et al., 2016).

The Troponin-I values were higher in the patients with physical stress, correlating to an increased incidence of clinical complications in this group. This finding indicates potential disturbances in coronary microcirculation; results of which have been discussed in other studies. TTC patients with physical stress were in general more critically ill than patients with emotional stress and comorbidities among patients was more pronounced in this group. A variant analysis of TTC showed no significant differences between the emotional and physical stress group.

We could further elucidate that the apical and non-apical variants, TTC with RV-involvement are manifestations of the same syndrome, differing significantly, in their clinical presentation, related complications and prognosis (Elesber et al., 2006; Haghi et al., 2006; Templin et al., 2015; El-Battrawy et al., 2016) and that catecholamines are secreted after stress induction and appear to have a central role in the pathophysiology of this syndrome.

## Conclusion

The presence of physical stress as a trigger factor was more frequent in male patients and correlated with significant serious short and long term complications. Further studies concerning the pathophysiological pathways are necessary to determine the differences in the outcomes of each group.

## Author Contributions

Conceived and designed the experiments: IE-B, KG, IA, and KS. Wrote the first draft of the manuscript: IE-B, KG, IA, and MB. Contributed to the writing of the manuscript: IE-B, IA, UH, and KG. Agree with manuscript results and conclusions: IE-B, IA, MB, and KS. Jointly developed the structure and arguments for the paper: IE-B, IA, KG, and KS. Made critical revisions and approved final version: MB, IE-B, and IA. All authors reviewed and approved of the final manuscript.

## Conflict of Interest Statement

The authors declare that the research was conducted in the absence of any commercial or financial relationships that could be construed as a potential conflict of interest.
