# Corrigendum: Comparison and Outcome Analysis of Patients with Takotsubo Cardiomyopathy Triggered by Emotional Stress or Physical Stress

**DOI:** 10.3389/fpsyg.2017.01114

**Published:** 2017-06-30

**Authors:** Konstantinos Giannakopoulos, Ibrahim El-Battrawy, Katja Schramm, Uzair Ansari, Ursula Hoffmann, Martin Borggrefe, Ibrahim Akin

**Affiliations:** ^1^First Department of Medicine, Faculty of Medicine, University Medical Centre Mannheim, University of HeidelbergMannheim, Germany; ^2^German Center for Cardiovascular Research (DZHK)Mannheim, Germany

**Keywords:** takotsubo cardiomyopathy, physical stress, emotional stress, prognosis, mortality

In the original article, the names of Konstantinos Giannakopoulos, Uzair Ansari, Martin Borggrefe, Ibrahim Akin were incorrectly spelled. The authors apologize for this error and state that this does not change the scientific conclusions of the article in any way.

The original article has been updated.

## Conflict of interest statement

The authors declare that the research was conducted in the absence of any commercial or financial relationships that could be construed as a potential conflict of interest.

